# Nanofibrous Membrane Dressings Loaded With Sodium Hydrogen Sulfide/Endothelial Progenitor Cells Promote Wound Healing

**DOI:** 10.3389/fbioe.2021.657549

**Published:** 2021-08-04

**Authors:** Jie Lian, Guanqun Ju, Xueyao Cai, Yuchen Cai, Chun Li, Sunxiang Ma, Yi Cao

**Affiliations:** ^1^Department of Plastic and Reconstructive Surgery, Shanghai Ninth People's Hospital, Shanghai Jiao Tong University School of Medicine, Shanghai, China; ^2^Department of Urology, Changzheng Hospital, Naval Medical University, Shanghai, China; ^3^Key Laboratory of Functional Genomic and Molecular Diagnosis of Gansu Province, Lanzhou, China

**Keywords:** sustained-releasing, H_2_S, nanofibrous membrane, endothelial progenitor cells, wound healing

## Abstract

Hydrogen sulfide (H_2_S) has been identified as an important gasotransmitter. H_2_S donor can release H_2_S sustained and is used as wound dressing. Endothelial progenitor cells (EPCs), given their regenerative ability, have also been reported to enhance wound healing. However, effective drug carriers are missing for the clinical application of H_2_S and EPCs. In this study, we investigated a novel drug carrier nanofibrous membrane, which was prepared by blending the recombinant spider silk protein (rMaSp) and sodium hydrogen sulfide (NaHS) by electrospun. Our results show that the rMaSp/NaHS nanofibrous membrane is associated with high hemocompatibility and cytocompatibility and is capable of stably releasing H_2_S for a long period of time. We also tested the rMaSp/NaHS membrane loaded with EPCs in an *in vivo* cutaneous wound model. We showed that the rMaSp/NaHS/EPC system significantly enhances wound regeneration efficiency as compared to rMaSp membrane and rMaSp/NaHS membrane. This study provides key evidence supporting the clinical application of nanofibrous membrane in the field of skin tissue regeneration.

## Introduction

Hydrogen sulfide (H_2_S), which plays important roles in inflammation, reperfusion injury, and circulatory shock of cardiovascular and nervous systems, can rapidly travel through the cell membranes to exert its biological effects, resulting in multiple cytoprotective responses, such as anti-inflammation, relaxing vascular, stimulating angiogenesis, and attenuating oxidative-stress-related tissue injuries (Wu et al., 2016). H_2_S protects fibroblast cells from H_2_O_2_-induced oxidative damage and promotes expressions of wound healing-related genes *in vitro* (Feng et al., 2015). *In vitro* and *in vivo* studies have shown that H_2_S promotes the proliferation and migration of vascular endothelial cells, the formation of microvessels, and wound healing (Papapetropoulos et al., 2009; Wallace et al., 2009; Altaany et al., 2013). As H_2_S precursors, sodium hydrogen sulfide (NaHS) or sodium sulfide (Na_2_S) has been used as an ingredient to prepare biomaterial for wound healing. Due to the multiple physiological functions of H_2_S, the biomaterials loaded with H_2_S donors capable of sustained release of H_2_S have a good application prospect. Because of the aforementioned physiological of H_2_S, biomaterials loaded with H_2_S donors have a good application prospect. Many studies have been using NaHS to release H_2_S in the presence of water. However, the lifetime of NaHS is short, making it difficult to maintain a constant cellular concentration of H_2_S over a prolonged period. In order to provide sustained releasing of H_2_S, it is necessary to find a suitable biomaterial as a carrier. Endothelial progenitor cells (EPCs) are vascular EPCs, which can secrete a variety of vascular factors to promote vascular repair and regeneration and can also participate in vascular regeneration (Asahara et al., 1997; Lin et al., 2000). Therefore, EPCs have been used to enhance wound healing (Kim et al., 2009; Asai et al., 2013).

Spider silk, known for their strength, toughness, good elasticity, and biocompatibility, can be used as promising biomaterials, showing a wide range of applications in the field of biotechnology and biomedicine (Omenetto and Kaplan, 2010). Among them, MaSp1, which is the main component protein of dragline silk with the best comprehensive performance, has attracted much attention in the research of medical materials. Because natural spider silk cannot be farmed due to the cannibalistic habits, strong territorial awareness, and limited silk production of most spiders, recombinant spider silk protein obtained from bioengineering served as an ideal substitute. It is worth mentioning that some functional amino sequences can be fused with the repetitive modules endowing the recombinant silk protein novel biomedical properties and expanding their applications in skin regeneration, and cell and tissue engineering (Andersson et al., 2017; Resch et al., 2018; Baklaushev et al., 2019). Furthermore, recombinant spider silk protein can be made from biomaterials with different processing methods, including electrospinning, which can prepare nanoscale fibers with high porosity, large specific surface area, high degree of fineness, and uniformity of fibers. Electrospun nanofibrous membranes from recombinant spider silk protein have high application potential in the biomedical field (Putzu et al., 2019).

In this study, the nanofibrous membrane was electrospun from the recombinant spider silk protein blended with NaHS as H_2_S donor, exhibiting sustained releasing of H_2_S. The membrane was loaded with EPCs and employed as a novel wound dressing system to enhance wound regeneration. The wound healing effects were assessed using a full-thickness cutaneous wound model with BALB/c male mice. We found that nanofibrous membrane-loaded NaHS/EPCs significantly enhanced wound healing by releasing H_2_S and EPCs.

## Materials and Methods

### Preparation of Recombinant Spider Silk Protein

The rMaSp and recombinant spider silk were obtained by the method as described in the previous literature. The construction of rMaSp is composed of a 6× His tag and six units consisting of two repeated polyalanine and glycine-rich regions derived from MaSp1 of *Elsinoë australis* (Andersson et al., 2017). In brief, the polyalanine- and glycine-rich regions were transferred into BL21 (DE3) *Escherichia coli* cells that were cultured overnight in Luria broth (LB) media at 37°C, 200 rpm. Then, the *E. coli* was then cultured in a 1/100 inoculation of 500 ml LB media at 30°C, 180 rpm, until OD_600_ reached 0.6–0.8. *E. coli* cells were finally induced by adding isopropylthiogalactoside to a concentration of 0.3 mM. Then, the *E. coli* cells were cultured overnight at 20°C, 180 rpm, and harvested by centrifugation for 20 min, 5,000 rpm at 4°C. The pellets were resuspended in 20 mM Tris pH 8 and lysated immediately with a sonicator. Then, the lysate was centrifuged at 12,000 rpm at 4°C for 30 min. Supernatants were loaded on a Ni-NTA column, and the protein was eluted with 300 mM imidazole. The eluted protein solution was dialyzed against 20 mM Tris pH 8, at 4°C overnight. SDS-PAGE (10%) and Coomassie Brilliant Blue staining were used to determine the protein purity.

### Electrospinning of Nanofibrous Membrane With H_2_S Donor

Hydrogen sulfide donor, NaHS, bought from Shandong Borman Chemical Co., Ltd., China. NaHS was dissolved in 1 mg/ml ethanol. Recombinant spidroin was dissolved in 1,1,1,3,3,3-hexafluoro-2-propanol (HFIP, Shanghai Jizhi Biochemical Technology Co., Ltd., China) to form an 8% w/v spinning dope, then different weights of NaHS (3.125, 6.25, 12.5, 25, and 50 μg/ml) were added to prepare samples with H_2_S donors, and finally, the concentration of rMaSp in all samples were adjusted to 10% (w/w). The nanofibrous membrane was obtained by a homemade electrospinning system through the stationary collector described earlier with a flow rate at 0.6 ml/h, voltage of 10 kV, and 50% humidity.

### Fiber Characterization

The morphology of the nanofibrous membrane was scanned with a Quanta FEG250 scanning electron microscopy (FEI, Michigen, USA). The fiber samples were dried under nitrogen flow and then coated with gold. Three areas were randomly selected to test the uniformity of the fibers in each sample.

To analyze the Fourier transform infrared spectroscopy (FTIR) spectrum of dried rMaSp membrane and rMaSp/NaHS membrane, the Nicolet 5700 Fourier transform infrared spectrometer was used with a spectral range of 4,000–676 cm^−1^, the scanning time of 32, and the resolution of 0.04 wavenumber/cm.

Hemolysis assays of the nanofibrous membrane were performed according to the previous literature and performed as follows: Before the experiment, strips of the nanofibrous membrane (10 × 30 mm) were sterilized in 75% ethanol for 0.5 h and then washed three times with sterile water. In brief, healthy red blood cells (HRBCs) were harvested from fresh white rabbit suspending in 3.8% sodium citrate (the ratio of sodium citrate to distilled water is 3.8:100 w/v) and centrifuged at 1,200 rpm for 10 min, to harvest serum-free HRBCs. The pure HRBCs were washed 5 times with phosphate-buffered saline (PBS) and then diluted 10 times in PBS before hemolysis assays. The diluted HRBCs (0.2 ml) were mixed with 10 ml deionized water as a positive control and 10 ml PBS buffer solution as a negative control. After gentle shaking, all nanofibrous membranes were incubated at 37°C for 2 h and centrifuged at 2,000 rpm for 5 min. The supernatant was carefully removed. The absorbance of the supernatant was measured at 545 nm using an UV-visible spectrophotometer (PerkinElmer, Massachusetts, USA). The formula for calculating the hemolysis ratio is as follows:

(1) $$\begin{array}{l} HR=\left(SA-NA\right)/\left(PA-NA\right)\times 100\% \end{array}$$

where *SA, PA*, and *NA* represent the absorbance of the experimental sample, positive control, and negative control, respectively. The assays were repeated three times to ensure reproducibility.

### Test the Mechanical Properties of Nanofibrous Membranes

The mechanical properties of nanofibrous membranes were examined by a universal material tester H5K-S (Hounsfield, England) with a crosshead speed of 30 mm/min and a gauge length of 30 mm. All the nanofibrous membranes were cut into strips (50 × 10 mm), in which four random strips of each fibrous membrane were tested to calculate the stress and strain of the nanofibrous membranes according to the previous literature (Hong et al., 2011).

### Detect the Wettability of the rMaSp or rMaSp/NaHS Membrane

Wettability of the nanofibrous membranes was determined from water contact angles measured with a drop shape analyzer Kyoma G-1 and the sessile drop technique. All data were analyzed with ADVANCE-drop shape software to determine the contact angles. Both membranes consist of three samples, and each sample test was repeated three times.

### Testing the Water Vapor Transmittance of the rMaSp or rMaSp/NaHS Membrane

The water vapor transmittance (WVTR) of the rMaSp or rMaSp/NaHS membrane in contact with water vapor was determined according to the pharmaceutical industry standard YY/T 0471.2-2004 of the People's Republic of China. The membrane film to be tested is encapsulated in the opening of the moisture permeable cup filled with deionized water, and the lower surface of the film is in contact with the opening of the cup. Then, it is placed in a constant temperature and humidity box with 37 ± 1°C and relative humidity (RH) of 20%. After a period of time, weigh the samples and measure the moisture permeability. There are five samples in each group, and the average value of the results is calculated by the following formula:

$$\begin{array}{l} WVTR\left(g/m^{2}/day\right)=\left(W1-W2\right)/A\times t\times 24\times 10^{6} \end{array}$$

where W1 is the mass before the test (g), W2 is the mass after the test (g), *A* is the effective area of the moisture permeable cup (mm^2^), and *t* is the test time (h).

### The Antibacterial Properties of rMaSp Membrane and rMaSp/NaHS Membrane

Soak a diameter of 1.5 cm of rMaSp membrane or rMaSp/NaHS membrane totally in 75% ethanol for 5 min, then dry the membranes, and put them on the agar plate. At the same time, 0.2 ml of *E. coli* or *Staphylococcus aureus* that cultured overnight was inoculated in the center of the membrane and cultured at 37°C for 7 days.

### Profile of H_2_S Release

Release kinetics of H_2_S from nanofibrous membrane was measured according to the previous study (Paul and Snyder, 2015; Lin et al., 2017). A total of 20 mg of membrane was immersed in 50 ml PBS (pH 7.4). To minimize the volatilization loss, the membranes were separately prepared and conducted for each test time point in 48 h. Then, the membranes were sealed with Tegaderm™ at 33°C and then withdrawn periodically at prescribed time intervals. Reaction aliquots (0.5 ml) were added to the mixture of zinc acetate (50 μL, 1% w/v in H_2_O) and NaOH (6.25 μL, 1.5 M) in 1.5 ml centrifuge tubes at certain time intervals. Then the solution was centrifuged at 20,500 RCF for 1 h, followed by removing the supernatant using a pipette. FeCl_3_ (100 μL, 30 mM in 1.2 M HCl) and *N, N*-dimethyl-*p*-phenylenediamine sulfate (100 μL, 20 mM in 7.2 M HCl) were added to the centrifuge tubes. Finally, the solutions were transferred into a 96-well microplate followed by the addition of 1 ml water, and the absorbance (670 nm) was taken after 20 min.

### Cell Lines and Cell Cultures

The NIH 3T3 cells of the mouse fibroblast cell line were purchased from the American Type Culture Collection, USA, and the mouse EPCs were purchased from the Shanghai Institute of Biochemistry and Cell Biology, Chinese Academy of Sciences, China. The cells were maintained in Dulbecco's Modified Eagle's Medium (DMEM, Sigma-Aldrich, Missouri, USA) supplemented with 10% heat-inactivated fetal bovine serum (HyClone, Thermo Scientific, Missouri, USA), 100 U/ml penicillin, and 100 μg/ml streptomycin (Gibco BRL, Invitrogen Corp., Carlsbad, CA, USA). They were cultured in a 5% CO_2_ humidified incubator at 37°C. Then, they were trypsinized and seeded on the sterilized rMaSp or rMaSp/NaHS nanofibrous membrane with a cell density of 5 × 10^3^ cells per cm^2^.

### Cell Viability Assay

CCK-8 assays were performed to evaluate the cytocompatibility of rMaSp or rMaSp/NaHS nanofibrous membranes. At each test time point, 12, 24, 48, and 72 h, the culture medium was removed, the membranes were washed with PBS, and the fresh medium was added into the wells. Then, the CCK-8 assays were operated according to the protocol of the manufacturer. Each measurement was repeated three times.

### Animal Cutaneous Model for Wound Healing

Male BALB/c mice (Jiesijie Experimental Animal Co., Ltd., China) weighing 20 ± 3 g were treated strictly in accordance with the International Ethical Guidelines and the National Institutes of Health Guide concerning the Care and Use of Laboratory Animals. The mouse was anesthetized with 4% chloral hydrate, and the skin was cleaned with the back skin exposed. Silicone rings with an internal diameter of 8 mm and a thickness of 0.5 mm were stitched on the skin. Two full-thickness wounds per mice were created on their mid-back with a 6-mm diameter puncher (Acuderm^®^ Inc., Ft Lauderdale, FL, USA).

For the rMaSp/NaHS/EPC group, all operations were performed under sterile conditions. First, the rMaSp/NaHS/EPC membrane was prepared on the sterile round cover glass with a diameter of 2 cm. Then, the cover glass was placed on the bottom of a 3-cm cell culture dish. Finally, 1 × 10^5^ EPCs were inoculated in each culture dish. After cultured for 48 h, the cover glass and the rMaSp/NaHS/EPC membrane were carefully separated with tweezers. The rMaSp/NaHS/EPC member was immediately transplanted to experimental animals. After the rMaSp/NaHS/EPC membrane covered the wound, the mice were sealed and bandaged with sterile gauze. Mice were fed in the sterile mouse feeding room, and EPCs can survive in the environment provided by the mouse subcutaneous tissue under aseptic conditions. The optical photographs of each wound were taken. RMaSp, rMaSp/NaHS (10% w/w NAHS to RMASP), and rMaSp/NaHS/EPC with a diameter of 7 mm were deposited in wound areas. Wounds were covered with medical bandages. After transplantation, the photos of general wound areas were taken on days 7, 14, and 20 and analyzed with the ImageJ software. The ratio of wound healing was calculated as follows:

(2) $$\begin{array}{l} C\left(\%\right)=\frac{C_{0}-C_{f}}{C_{0}}\times 100\% \end{array}$$

where *C*% is the wound healing closure ratio, *C*_0_ is the original wound area, and *C*_f_ is the open area on point day.

### Hematoxylin–Eosin Staining

The skin histological analysis was performed according to the previous literature on days 7 and 20 after transplantation. After anesthesia of the mice, the wound skin tissues were excised, maintained in cold 4% paraformaldehyde in 0.01 M PBS (pH = 7.4) overnight, embedded in paraffin, and cut into 5-μm thick sections with microtomes (LEICA RM2235, Germany). Four wound skin areas were analyzed at each time point.

The wound skin was stained with hematoxylin and eosin (H&E) (Beyotime Institute of Biotechnology, China) for morphological evaluation. The sections were put in the xylene for 20 min, 100% alcohol for 5 min, 95% alcohol, and 80% alcohol for 2 min, distilled water for 5 min, hematoxylin nuclear stain for 5 min, and PBS washed for 3 min to remove the extra H&E staining in the extracellular matrix for 2 min. Then, the slices were washed with deionized water for 5 min, the sections were dehydrated through increasing concentration of ethanol and xylene, and sealed with neutral resin. The photographs were taken with a Nikon microscope (Nikon, Tokyo, Japan).

### Evaluation of Deposit Collagen on Wound Skin

Collagen deposition was measured by the insoluble collagen assay according to the previous literature (Wirohadidjojo et al., 2011). A 5-mg tissue of each wound skin was cut from the wound areas and put in a 96-well plate. After washed with PBS, the tissues were fixed with Bouin solution for 1 h and rinsed with tap water until all the yellow color was completely removed. Then the tissues were dried at room temperature overnight. A 200-μL Sirius Red diluted in saturated picric acid was added for 1 h. Then the unbinding Sirius Red was washed with 200 μL of 0.1 N HCl. Sirius Red on the well bases was then removed by dilution with 200 μL of 0.5 N NaOH. The tissue slides were observed at 550 nm.

### Statistical Analysis

All data were expressed as mean ± SD. The statistical differences were performed using one-way ANOVA followed by Tukey's test with GraphPad Prism 6 software (GraphPad Software Inc., La Jolla, CA, USA). For all tests, ^*^ indicates *p* value < 0.05, ^**^ *p* value < 0.01, and ^***^ *p* value < 0.001.

## Results

### Preparation and Characterization of Nanofibrous Membrane

**Figure 1:**
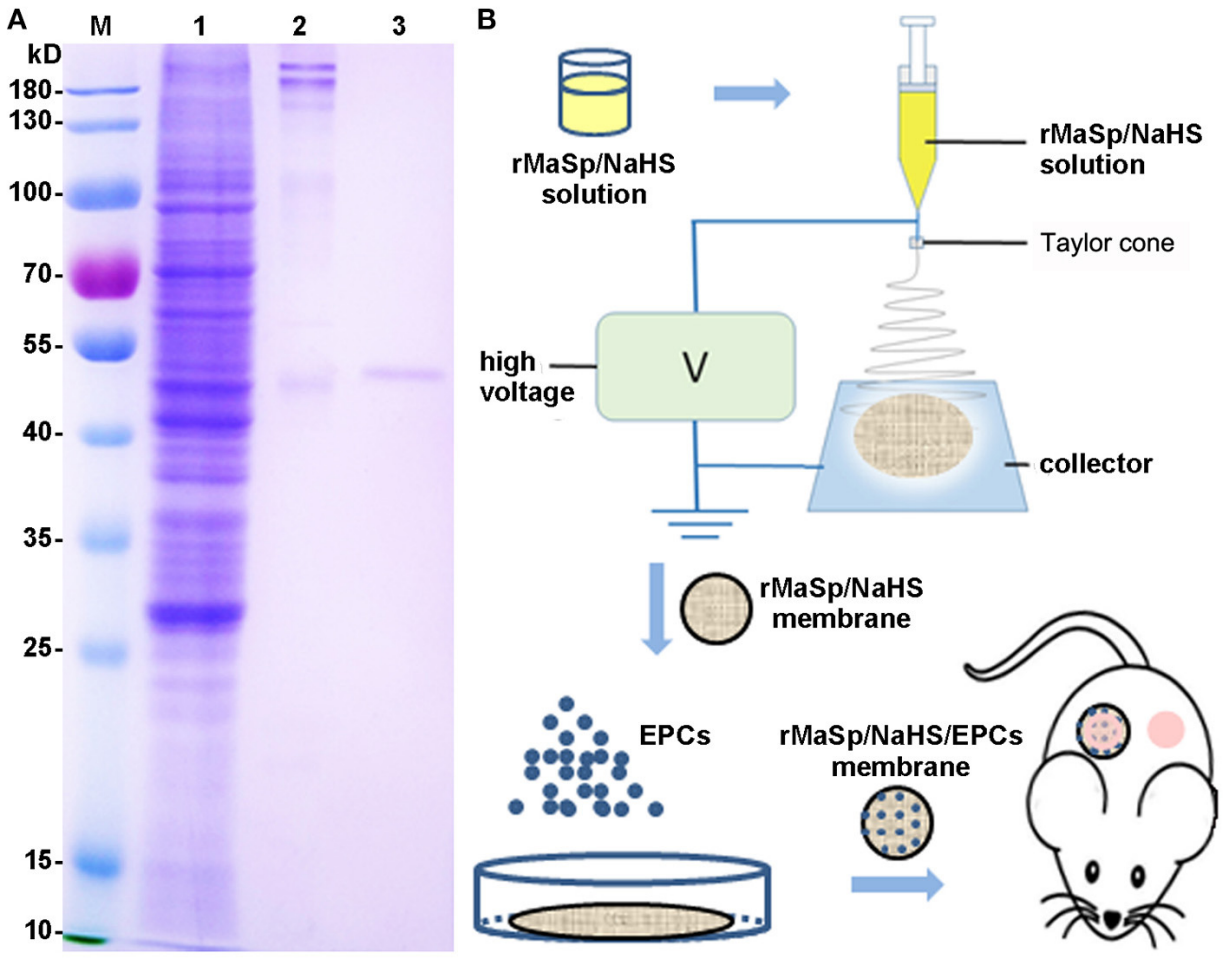
Preparation of recombinant spider silk protein (rMaSp) and the schematic diagram of wound healing program. **(A)** Coomassie Brilliant Blue staining of rMaSp purification. Lanes of w1–3 are protein ladder, supernatant of lysate, precipitation of lysate, rMaSp, respectively. **(B)** The schematic diagram of wound healing program with rMaSp/sodium hydrogen sulfide (NaHS)/endothelial progenitor cell (EPC) membrane.

The recombinant spider silk protein, rMaSp, consists of six units containing two polyalanine and glycine-rich repeated regions from MaSp1 of *E. australis*. Its molecular weight is 50 kDa, and the amino acid sequence is listed in Supplementary Figure 1. rMaSp was successfully expressed in *E. coli* BL (21DE3) cells and purified with an affinity chromatography processing. The result of Coomassie Brilliant Blue staining (Figure 1A) showed that the purity of the interested protein was above 95%.

**Figure 2:**
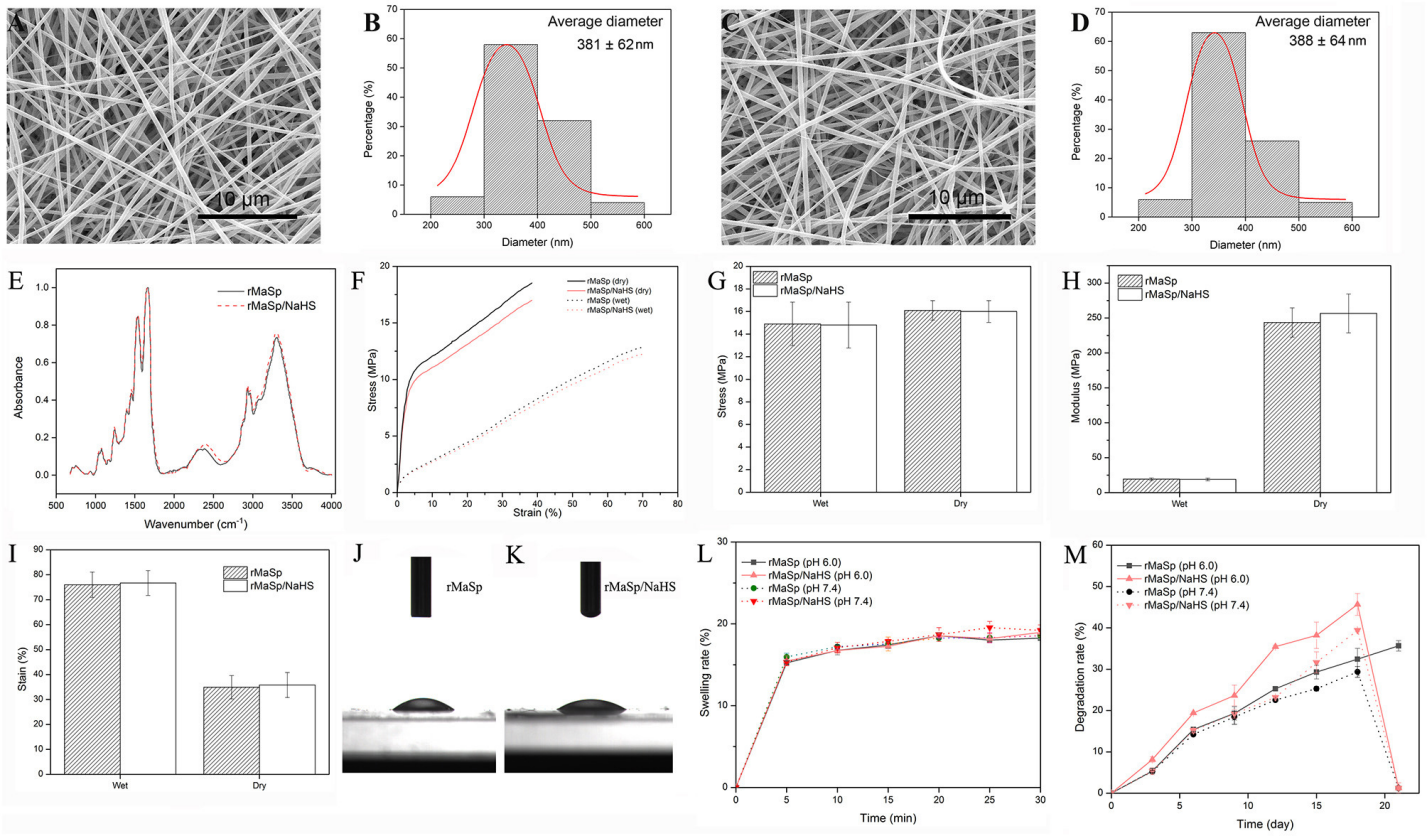
Characterization of nanofibrous membrane. The SEM images of rMaSp **(A)** and rMaSp/NaHS membrane **(C)** and diameter distribution of rMaSp **(B)** and rMaSp/NaHS nanofibers **(D)**. The Fourier transform infrared spectroscopy spectrum of dried rMaSp membrane and rMaSp/NaHS membrane **(E)**. The mechanical properties of dry and wet rMaSp/NaHS membrane **(F–I)**. The water droplets on the rMaSp **(J)** or rMaSp/NaHS membrane surfaces **(K)**. The swelling capacity of rMaSp or rMaSp/NaHS membrane **(L)**. The degradation profile of rMaSp or rMaSp/NaHS membrane **(M)**.

According to the characteristics of compatibility (Supplementary Figure 2), porosity (Supplementary Figure 3), and water vapor transmission rate (WVTR) (Supplementary Figure 4) of EPCs, the protein solution spinning system with different concentrations of NaHS was screened, and the protein solution spinning system with 12.5 μg/ml NaHS was finally selected for subsequent experiments. rMaSp and rMaSp blended with NaHS were fabricated into rMaSp membrane and rMaSp/NaHS membrane with an electrospinning process as shown in Figure 1B. Figures 2A,C showed the SEM image of rMaSp and rMaSp/NaHS, Figures 2B,D showed diameter of rMaSp and rMaSp/NaHS. According to the results, both rMaSp and rMaSp/NaHS generated smooth nanofibers. The diameters of rMaSp and rMaSp/NaHS were 381 ± 62 nm and 388 ± 64 nm, which were similar to the fibers generated from pure rMaSp solution. The Nicolet 5700 Fourier transform infrared spectrometer was used to analyze the FTIR spectrum of dried rMaSp membrane and rMaSp/NaHS membrane, and the results are shown in Figure 2E.

Figures 2F–I show the mechanical properties of dry and wet membranes. We also found that the stress of membranes dropped and strain increased due to the absorbance of water. Although the water changed the mechanical properties of the membrane, the stress and strain were still in the range of skin mechanical properties (i.e., stress 5–30 MPa, strain 35–115%).

The wettability has been reported to have great influences on the initial adhesion and proliferation of cells (Birky, 1989; Wei et al., 2009). The dynamic water contact angle was visualized to measure the hydrophilicity. The water droplets on the membrane surfaces were shown in Figures 2J,K. Both the membranes of rMaSp and rMaSp/NaHS showed a hydrophobic surface with the water angle of 20.07 and 21.26, respectively. The swelling capacity of rMaSp and rMaSp/NaHS membranes is shown in Figure 2L, and the results revealed that the swelling capacity of rMaSp membrane was similar to that of rMaSp/NaHS membrane at pH 6.0 and pH 7.4 PBS buffer, respectively. The degradation profiles of rMaSp and rMaSp/NaHS membranes were shown in Figure 2M, and the degradation of rMaSp membrane at pH 6.0 and pH 7.4 was similar to that of rMaSp/NaHS membrane at pH 7.4. However, the rMaSp/NaHS membrane at pH 6.0 has a relatively faster degradation rate, and this may be because of the faster H_2_S release rate.

### Sustained-Release Profile of H_2_S

**Figure 3:**
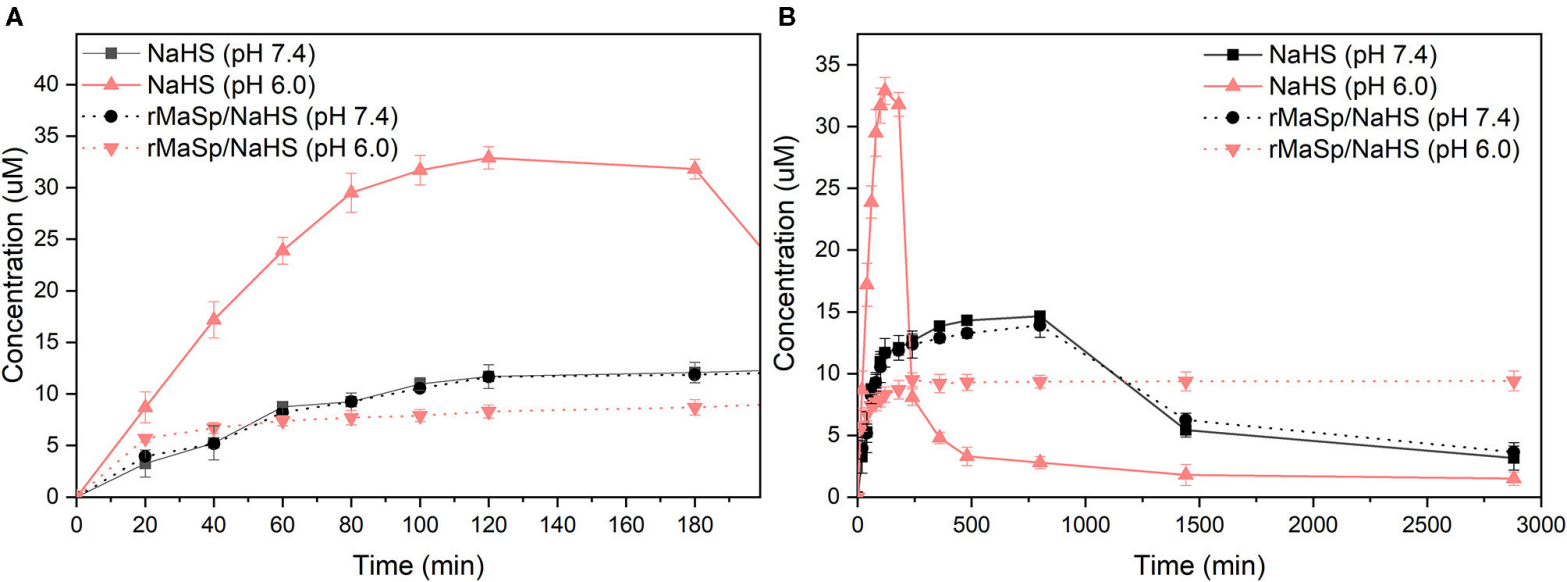
H_2_S releasing kinetics of donor NaHS vs. rMaSp/NaHS fiber (with 10% NaHS) under pH 7.4. **(A)** Short-term releasing profile (0–180 min). **(B)** Longer-term releasing profiles (0–2,880 min).

Hydrogen sulfide has been identified as the third member of the gasotransmitter, and it plays an essential physiological role in regulating the cytoprotective signal process (Shatalin et al., 2011). Under physiological environments, NaHS quickly reacts with water and releases H_2_S to elicit physiological responses; however, the short half-life of H_2_S limits its therapeutic potential (Kashfi and Olson, 2013). It would be a promising therapeutic strategy in biomedical applications to fabricate biocompatible scaffolds releasing H_2_S in a controlled manner. The releasing kinetics of H_2_S from the rMaSp/NaHS nanofibrous membrane (with 10% NaHS) was performed under pH 7.4 with NaHS in solution as the control. Longer-term and short-term releasing profiles (0–28,800 min) are shown in Figure 3. The curve of NaHS showed a burst release of H_2_S within 3 h. That is because, upon hydrolysis, NaHS dissociated and rapidly generated a large amount of H_2_S, leading to an instantaneously maximum concentration of H_2_S and then exponentially falling thereafter, likely owing to H_2_S loss from solution *via* volatilization and H_2_S oxidation in the physiological buffer (Lin et al., 2017). Unlike the releasing kinetics of NaHS donor in solution, the rMaSp/NaHS membrane showed a very mild burst of H_2_S with low concentration within 8 h, followed by a prolonged duration for 48 h. The instantaneous concentration of H_2_S was around 9 μM, which was covered in the range of 2–10 μM, the endogenous H_2_S plasma concentrations in healthy animals (Whitfield et al., 2008; Shen et al., 2011). The analytical results show that, by employing rMaSp, the duration of H_2_S release can be prolonged, increasing the potential applications.

### Biocompatibility of Nanofibrous Membrane

**Figure 4:**
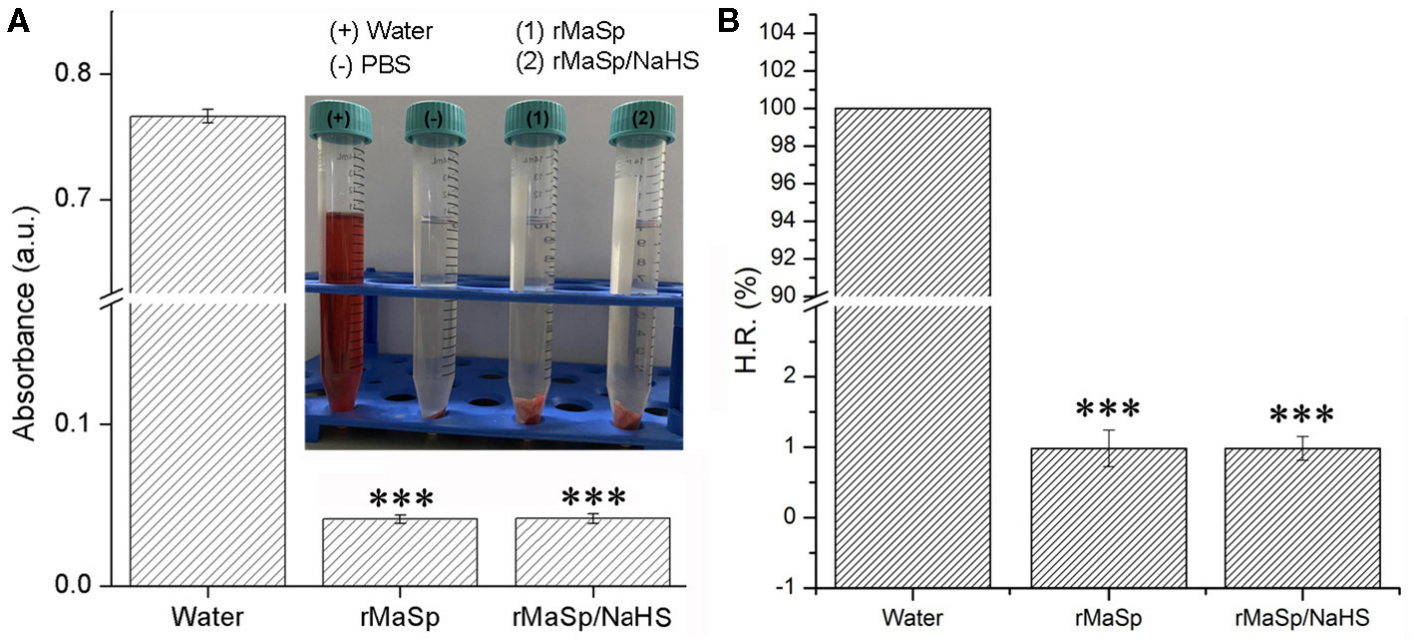
Hemolysis assays of rMaSp membrane and rMaSp/NaHS membrane. **(A)** The absorbance of each group in hemolysis assays was tested by the use of an absorbance photometer and **(B)** the hemolysis rate of rMaSp membrane and rMaSp/NaHS membrane. ^***^p < 0.001 compared to control group, *n* = 6.

Hemocompatibility and cytocompatibility of biomaterials codetermine the success of tissue engineering applications (Stoll et al., 2017). The results of hemolysis assays are shown in Figure 4, and the hemolysis rate of rMaSp membrane and rMaSp/NaHS membrane is 0.96 and 1.00%, respectively, which is far lower than 5%, demonstrating excellent hemocompatibility.

**Figure 5:**
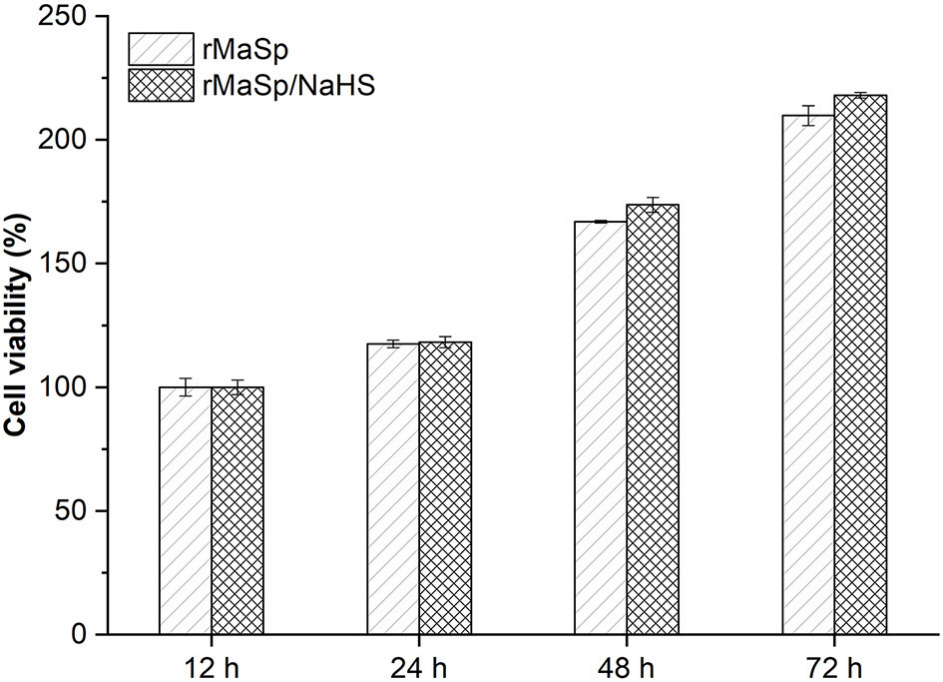
NIH 3T3 cell viability tests when cells were cultured on rMaSp membrane and rMaSp/NaHS membrane for 12–72 h, showing no significant difference in cell viability.

NIH 3T3 cells were cultured on the membranes for 72 h to investigate the cytocompatibility of rMaSp and rMaSp/NaHS membranes. Figure 5 shows that there is no significant difference in cell viability between the cells cultured on rMaSp membrane and rMaSp/NaHS membrane at each time point. Previous studies demonstrated that the rMaSp/NaHS membrane was non-toxic to fibroblast cells, which is essential for the wound healing process (Schacht et al., 2015). The RMaSp/NAHS membrane was assumed to maintain its capacity to support fibroblast cell proliferation *in vivo*.

### Endothelial Progenitor Cells Cultured on rMaSp/NaHS Membrane

**Figure 6:**
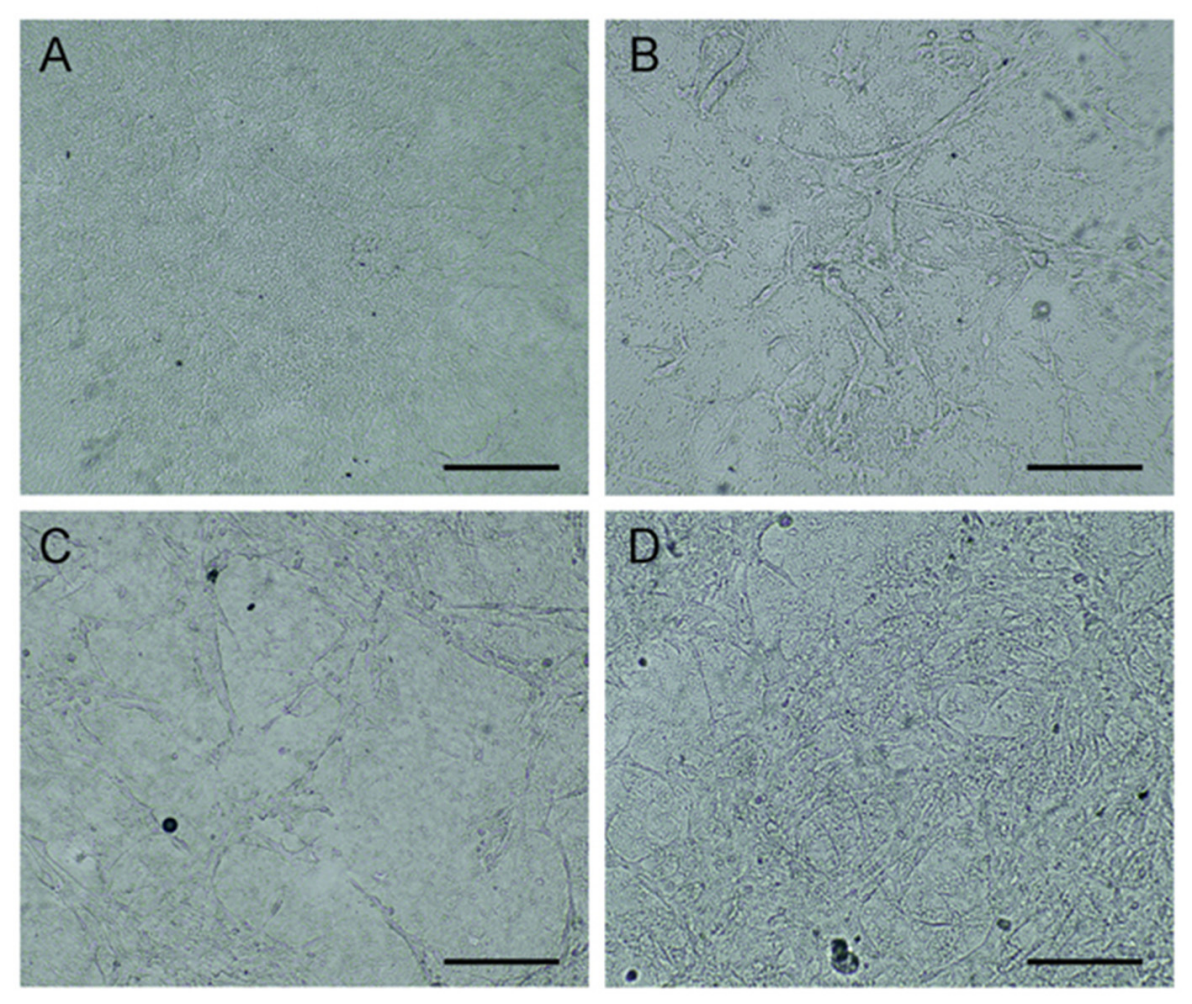
Endothelial progenitor cells (EPCs) cultured on rMaSp/NaHS membrane till the density of 90%. **(A)** Optical microphotographs of rMaSp/NaHS membrane. **(B–D)** EPCs cultured on rMaSp/NaHS membrane on days 1, 3, and 7, respectively.

Endothelial progenitor cells have been found that they can be used to enhance wound healing (Kim et al., 2009; Asai et al., 2013). In this study, EPCs were cultured on the rMaSp/NaHS membrane to form a novel wound dressing, rMaSp/NaHS/EPCs. Figure 6 shows the optical microphotographs of EPCs cultured on the rMaSp/NaHS membrane to the density of 90% within 7 days.

### *In vivo* Wound Healing

**Figure 7:**
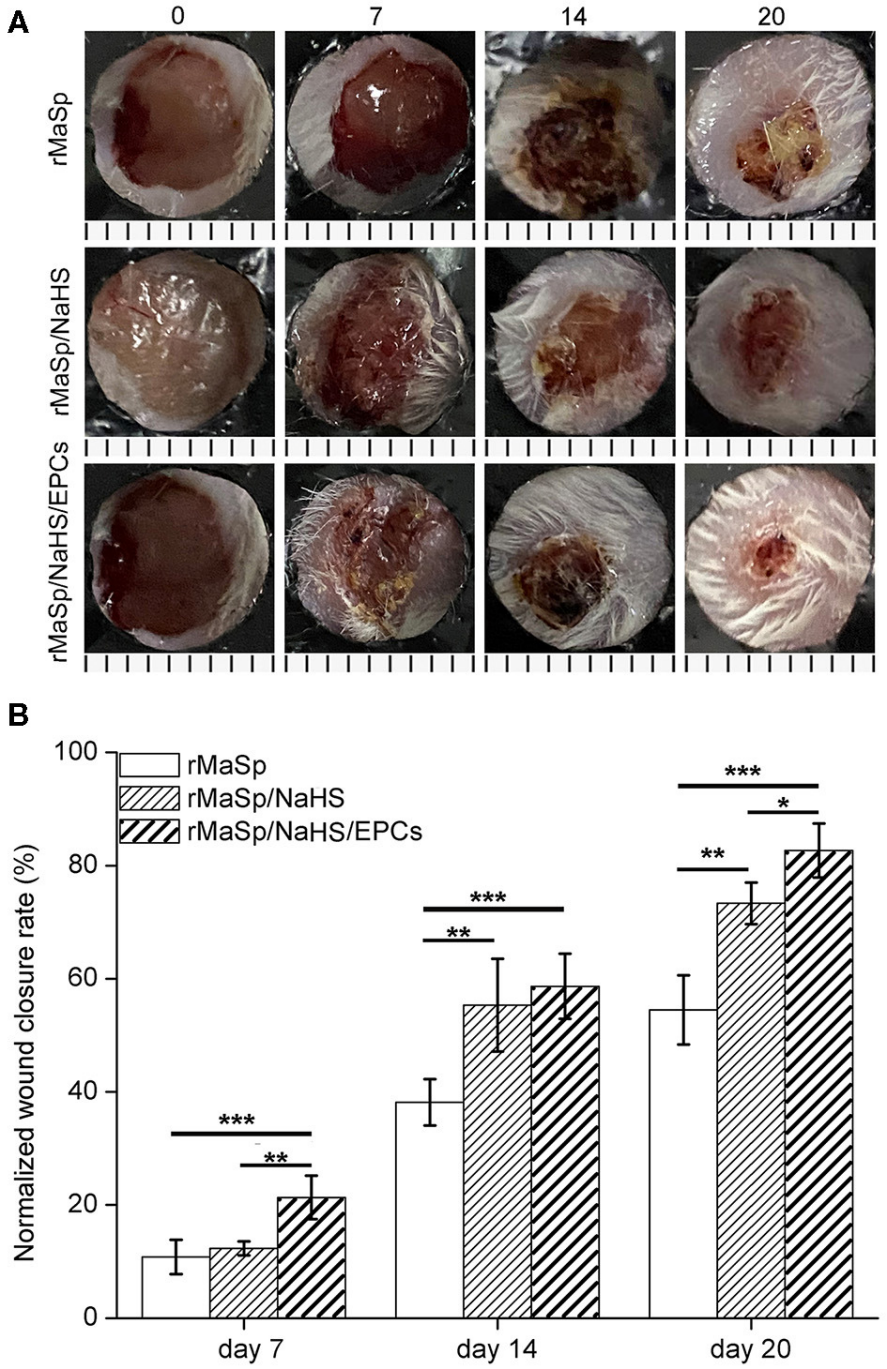
Wound closure upon treating with rMaSp membrane, rMaSP/NaHS membrane, and rMaSP/NaHS/EPC membrane. **(A)** Macroscopic observation of wounds covered with membranes on days 0, 7, 14, and 20. The unit of the rulers under the pictures is mm. **(B)** Wound closure rates of treated wounds. The statistical differences were performed using ANOVA. *p < 0.05, **p < 0.01, ***p < 0.001 compared with the control group, *n* = 6.

The wound healing efficacy of this novel H_2_S releasing and EPC-loaded nanofibrous membrane was evaluated by performing *in vivo* experiments on animal models. Full-thickness removal skin caused by cutaneous wounds in male BALB/c mice was established to study the wound healing capability. The wound healing progress was analyzed at different time points during 20 days after transplantation. Figure 7A shows the sequential macroscopic images of full-thickness models treated with rMaSp, rMaSp/NaHS, and rMaSp/NaHS/EPC nanofibrous membranes after days 7, 14, and 20, respectively. It can be seen that wounds were gradually regenerated from the edge of wound. Compared with the rMaSp membrane, rMaSp/NaHS/EPC-treated wounds showed enhanced wound closure at each time point, while the rMaSp/NaHS membrane-treated wounds were enhanced after 7-day treatment. These different results between rMaSp/NaHS membrane and rMaSp/NaHS/EPC suggested the positive function of the rMaSp/NaHS/EPC dressing likely due to the H_2_S releasing together with the loading EPCs. Figure 7B shows the quantitatively calculated wound closure rates for rMaSp-, rMaSp/NaHS-, and rMaSp/NaHS/EPC-treated wounds. Consistent with the visual macroscopic images of wounds, the healing rate of rMaSp/NaHS/EPC-treated group was significantly higher than that of rMaSp and rMaSp/NaHS at all the time points studied (day 7, 10, 14, and 17). Especially at day 20, the final closure rate for rMaSp/NaHS/EPC-treated group was 28, 9% faster than rMaSp- and rMaSp/NaHS-treated wounds, with a wound closure rate of 82.7 ± 4.8% in contrast to 54.5 ± 6.1 and 73.3 ± 3.7%. Both the macroscopic observation and quantified wound closure rate revealed that the healing of the wound was significantly improved under treatment with H_2_S releasing fibers together with loaded EPCs compared with only H_2_S releasing fibers.

**Figure 8:**
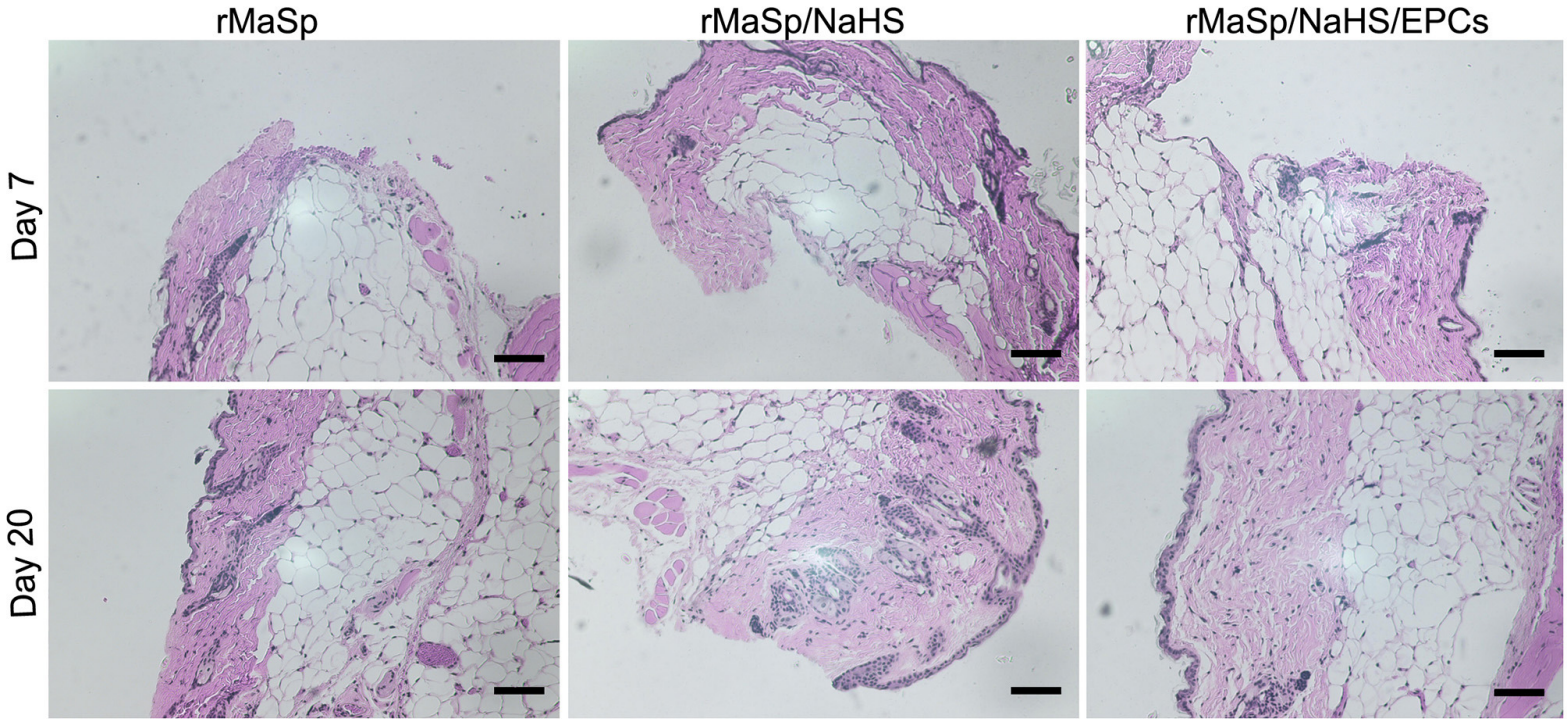
Hematoxylin and eosin (HandE) staining of wound site on days 7 and 20. Quantified granulation formation calculated from HandE staining on RMASP vs. RMASP-JK1 on days 7 and 20. Quantified reepithelization calculated from cytokeratin staining on RMASP vs. RMASP-JK1 on days 7 and 20. The statistical differences were performed using ANOVA. **p < 0.01, *p < 0.05 compared with the RMASP group, *n* > 3. The scale bar is 500 μm.

Cutaneous wound healing in adult mammals is a complex multistep process involving overlapping stages of blood clot formation, inflammation, reepithelialization, granulation tissue formation, neovascularization, and remodeling. As shown in Figure 8, the representative H&E staining histological images of granulation tissue are used to evaluate wound healing; Compared to rMaSp group, rMaSp/NaHS could get better regeneration of skin wound in mice, which may be due to slowly releasing of the H2S. Additionally, EPCs could promote angiogenesis in the wound, which has been supported by the histopathologic analysis. As shown in Figure 8, the effect of rMaSp/NaSH/EPCs promoting tissue regeneration was significantly higher than that of the other two groups, and the histopathologic analysis also supported these findings. The underlying dermis and other subcutaneous tissue were infiltrated by abundant granulation tissue. The complete and thick epidermis was seen in the rMaSp/NaHS/EPC-treated defects on day 21 in H&E staining images.

**Figure 9:**
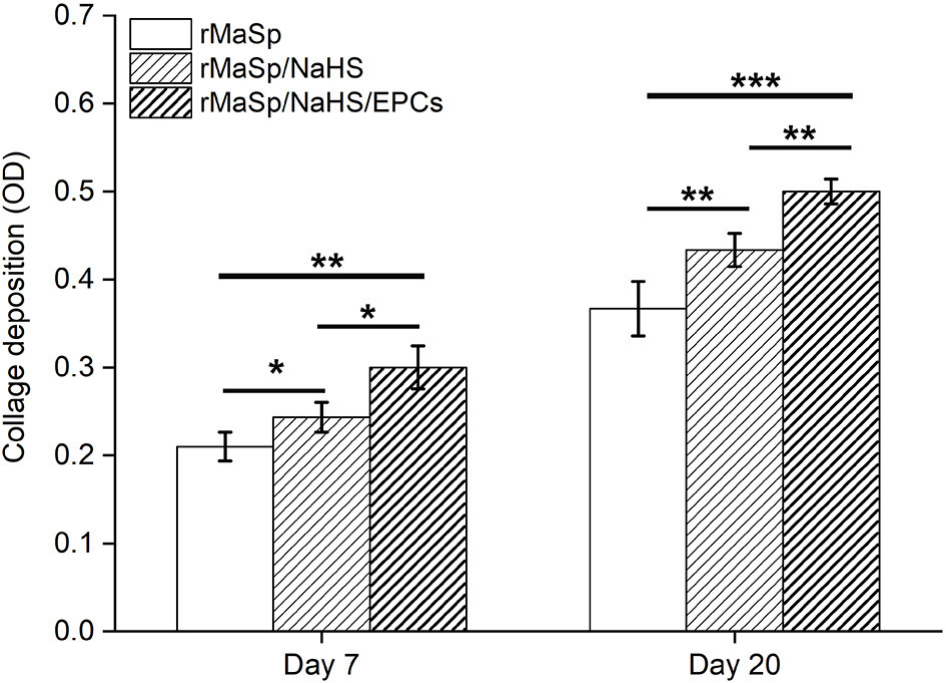
Collagen deposition upon post wound detected on days 7 and 20. ^*^p < 0.05, ^**^p < 0.01, ^***^p < 0.001 compared with the control group, *n* = 6.

Matrical collagen deposition upon post wound was quantitatively evaluated on day 7 and day 20. Figure 9 depicts the detection results of collagen deposition in regenerated skin at indicated time intervals. Deposition of collagen in rMaSp/NaHS-treated group and rMaSp/NaHS/EPCs-treated group is significantly higher than in rMaSp-treated group on both days 7 and 20. The deposition of collagen in rMaSp/NaHS/EPC-treated group is significantly higher than in rMaSp/NaHS-treated group. The results indicated that sustained releasing of H_2_S from nanofibers together with loading EPCs obviously enhanced neo-tissue formation.

## Discussion

Skin is the largest organ of the human body, which plays an important role as a barrier in protecting the body from the surrounding environment (Barros Almeida et al., 2021). The development of skin dressings to replace normal human skin is of great significance to the repair of human skin injury, where the dressings not only can protect the wound but also add the growth factor and other components to the skin dressing to promote wound healing (Yildirimer et al., 2012). Many studies reported that EPCs were recruited into injured tissues and contributed to neovessel formation by directly incorporating into vessel walls or by secreting a variety of angiogenic growth factors and subsequently stimulating angiogenesis (Rehman et al., 2003); both angiogenesis and vasculogenesis may play major roles in the process of neovascularization during adult wound healing (Kaushik and Das, 2019); and studies reported that EPCs were used to enhance the wound healing as seeding cell in tissue engineering (Kim et al., 2009; Asai et al., 2013). Maintaining the cell activity of EPCs is one of the key issues while repairing skin injury based on EPCs. Many studies verified that H_2_S is a potential gasotransmitter upon wound regeneration as it could promote proliferation and migration of EPCs, and meanwhile, H_2_S can promote microvessel tube formation as well as angiogenesis through the vascular endothelial growth factor receptor-2 (VEGF-2) pathway (Papapetropoulos et al., 2009; Coletta et al., 2012). NaHS has been widely used as the H_2_S donor (Kashfi and Olson, 2013), but the short-term burst from NaHS and short half-life of H_2_S hindered the application of NaHS, and it is necessary to prepare a biocompatible carrier that can release NaHS and H_2_S in a slow and prolonged manner.

In this study, a nanofibrous membrane, which is prepared by using recombinant spider silk protein (rMaSp) and NaHS, was contrasted by the use of electrospun. According to the results, the rMaSp/NaHS/EPC membrane prepared in this study has good biocompatibility and good mechanical strength. The *in vivo* experiment showed that it could adhere to the wound evenly and tightly for at least 3 weeks. The rMaSp/NaHS/EPC membrane not only can prevent bacteria from passing through but also has good air permeability. It can also release H_2_S stably for a long time. After loaded with EPCs, rMaSp/NaHS/EPC membrane was used as a wound dressing in a cutaneous wound model *in vivo*. The results showed that rMaSp/NaHS/EPC membrane could significantly enhance the wound regeneration compared with rMaSp membrane and rMaSp/NaHS membrane. Those results indicated that rMaSp/NaHS/EPC membrane wound dressing prepared in this study has a broad application prospect in the field of skin tissue regeneration.

## Conclusions

In this study, a novel wound dressing rMaSp/NaHS/EPC was designed, and its efficacy of wound healing was evaluated by using *in vivo* experiments. This study demonstrated that rMaSp/NaHS/EPCs could promote wound healing efficiency through H_2_S and EPCs. This study mainly focused on the effects of the constructed membrane structure on the proliferation of EPCs, the release properties of H_2_S, and the repair of skin injury in mice. The antibacterial properties and anti-inflammatory of the membrane are the limitations in this study, and they will be discussed in our future studies.

## Data Availability Statement

The original contributions presented in the study are included in the article/Supplementary Material, further inquiries can be directed to the corresponding authors.

## Ethics Statement

The animal study was reviewed and approved by the Ethical Board of Shanghai Ninth People's Hospital.

## Author Contributions

JL and GJ designed the research and performed the experiments. XC and YCai interpreted the results of the experiments. CL analyzed the data and prepared figures. SM and YCao designed the experiments, provided the experimental insight, and edited the manuscript. All authors read and approved the final manuscript.

## Conflict of Interest

The authors declare that the research was conducted in the absence of any commercial or financial relationships that could be construed as a potential conflict of interest.

## Publisher's Note

All claims expressed in this article are solely those of the authors and do not necessarily represent those of their affiliated organizations, or those of the publisher, the editors and the reviewers. Any product that may be evaluated in this article, or claim that may be made by its manufacturer, is not guaranteed or endorsed by the publisher.
