# Supplementary material for: Nanofibrous Membrane Dressings Loaded With Sodium Hydrogen Sulfide/Endothelial Progenitor Cells Promote Wound Healing
Source: Front Bioeng Biotechnol. 2021 Aug 4;9:657549. doi: 10.3389/fbioe.2021.657549 (PMC8372243; doi:10.3389/fbioe.2021.657549)
Supplement: Supplementary file 1 [file Table_1.DOC]

**Table S1. The antibacterial properties of rMaSp membrane and rMaSp/NaHS membrane**

|  | E. coli | Staphylococcus aureus |
| --- | --- | --- |
| rMaSp membrane | no penetration | no penetration |
| rMaSp/NaHS membrane | no penetration | no penetration |

**
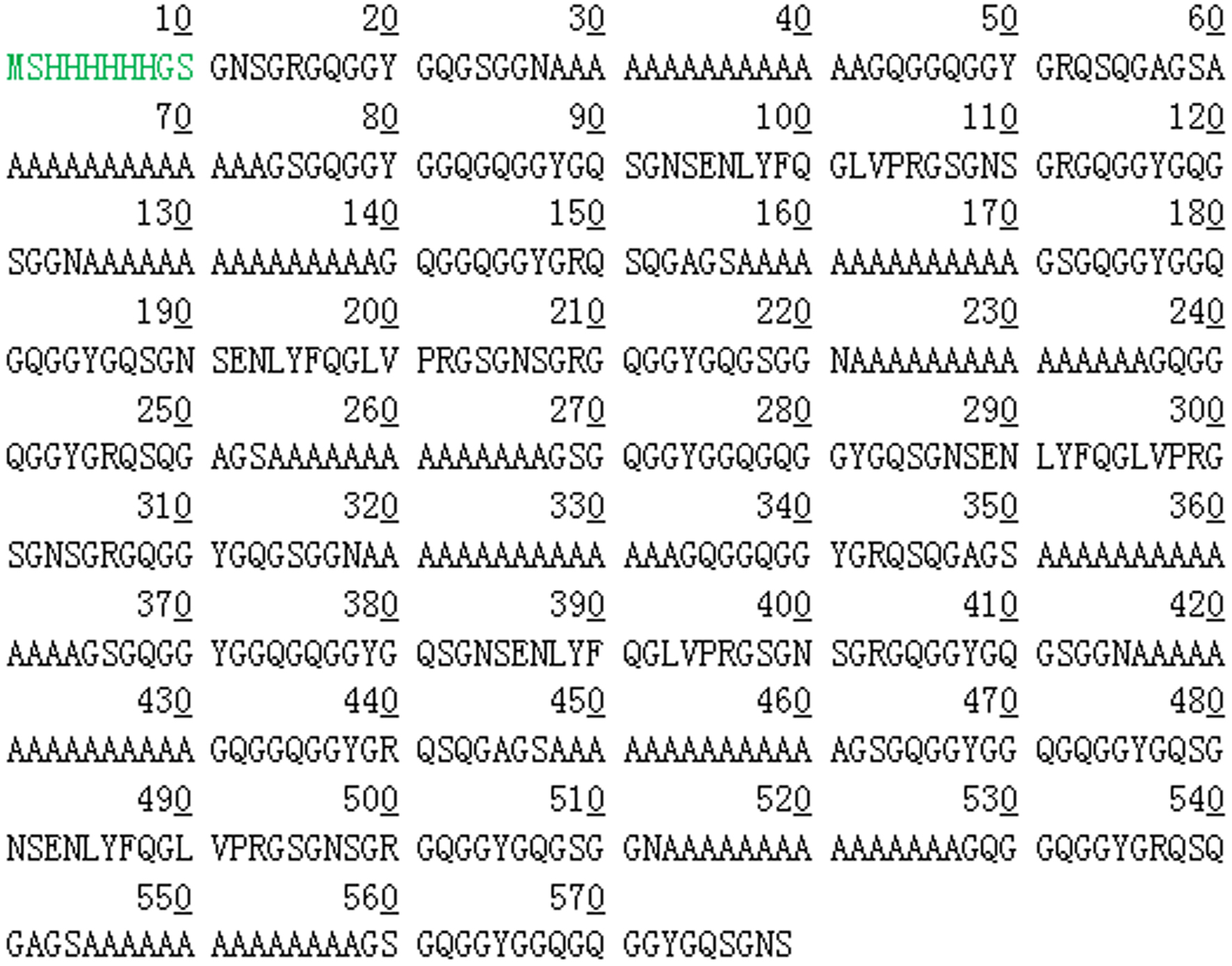
**

**Figure S1.** Amino acid sequence of rMaSp.


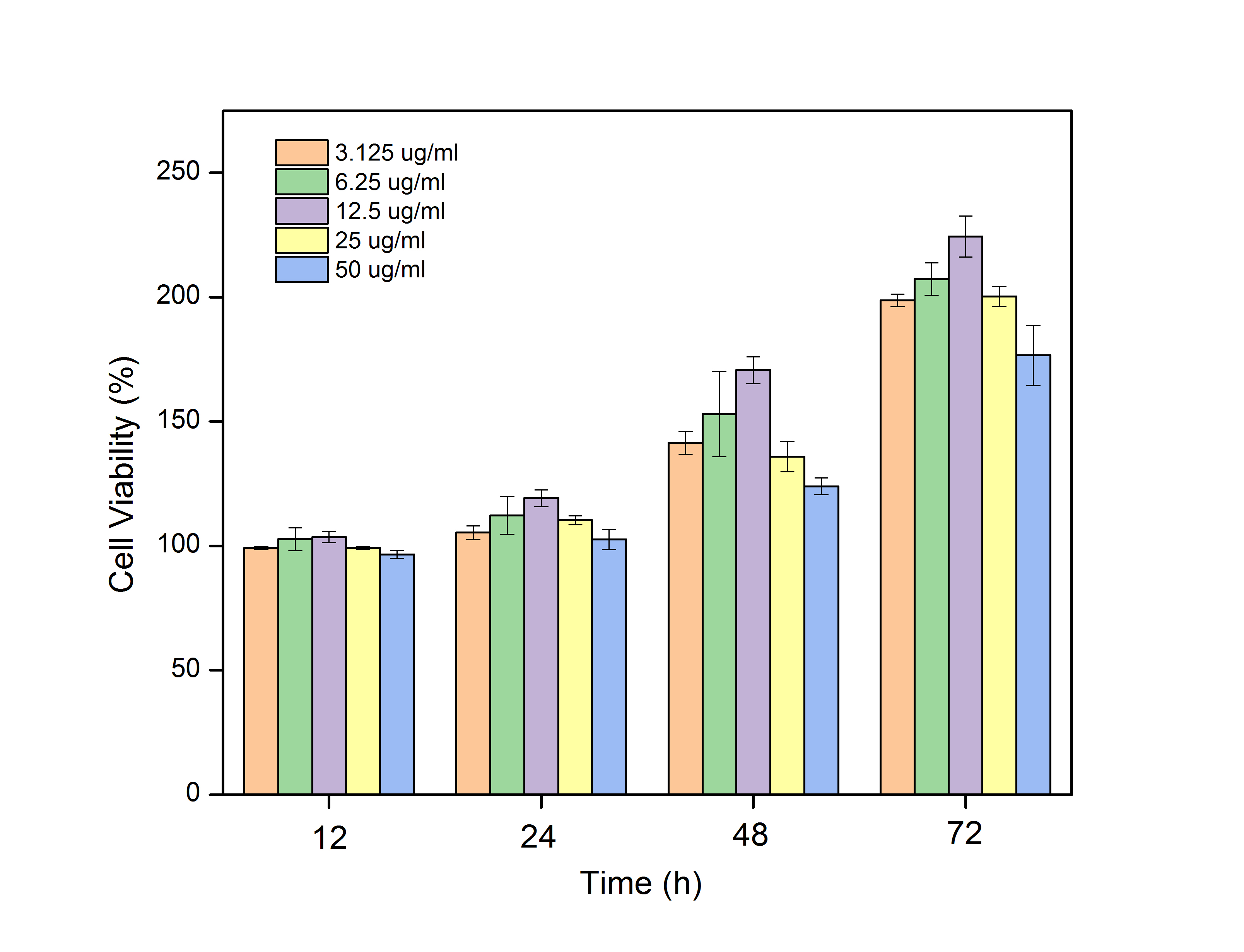
**Figure S2.** The effect of rMaSp/NaHS membrane with different concentration of NaHS on the cell viability of EPVs.


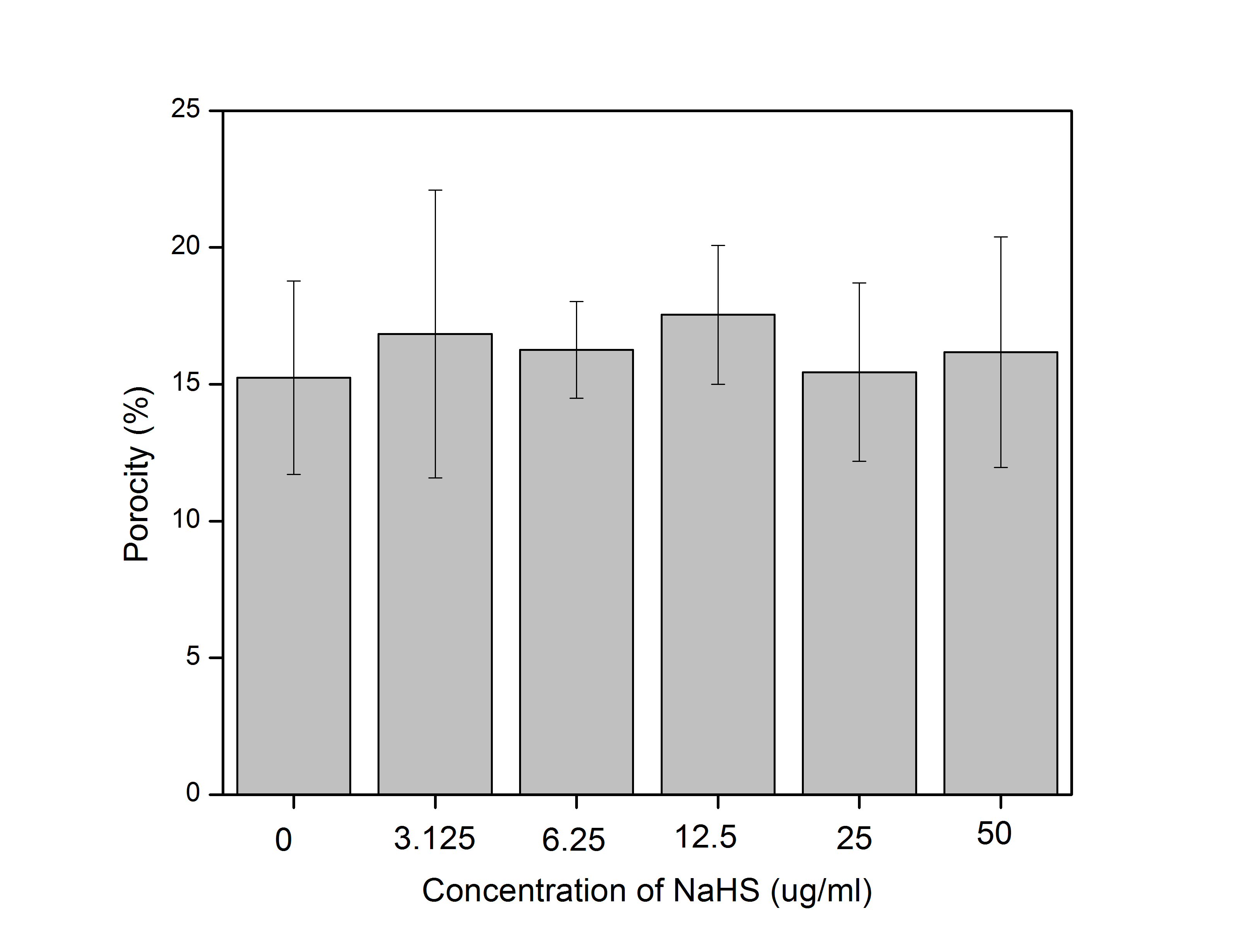


**Figure S3.** The porosity of the rMaSp/NaHS membrane with different concentration of NaHS.

**
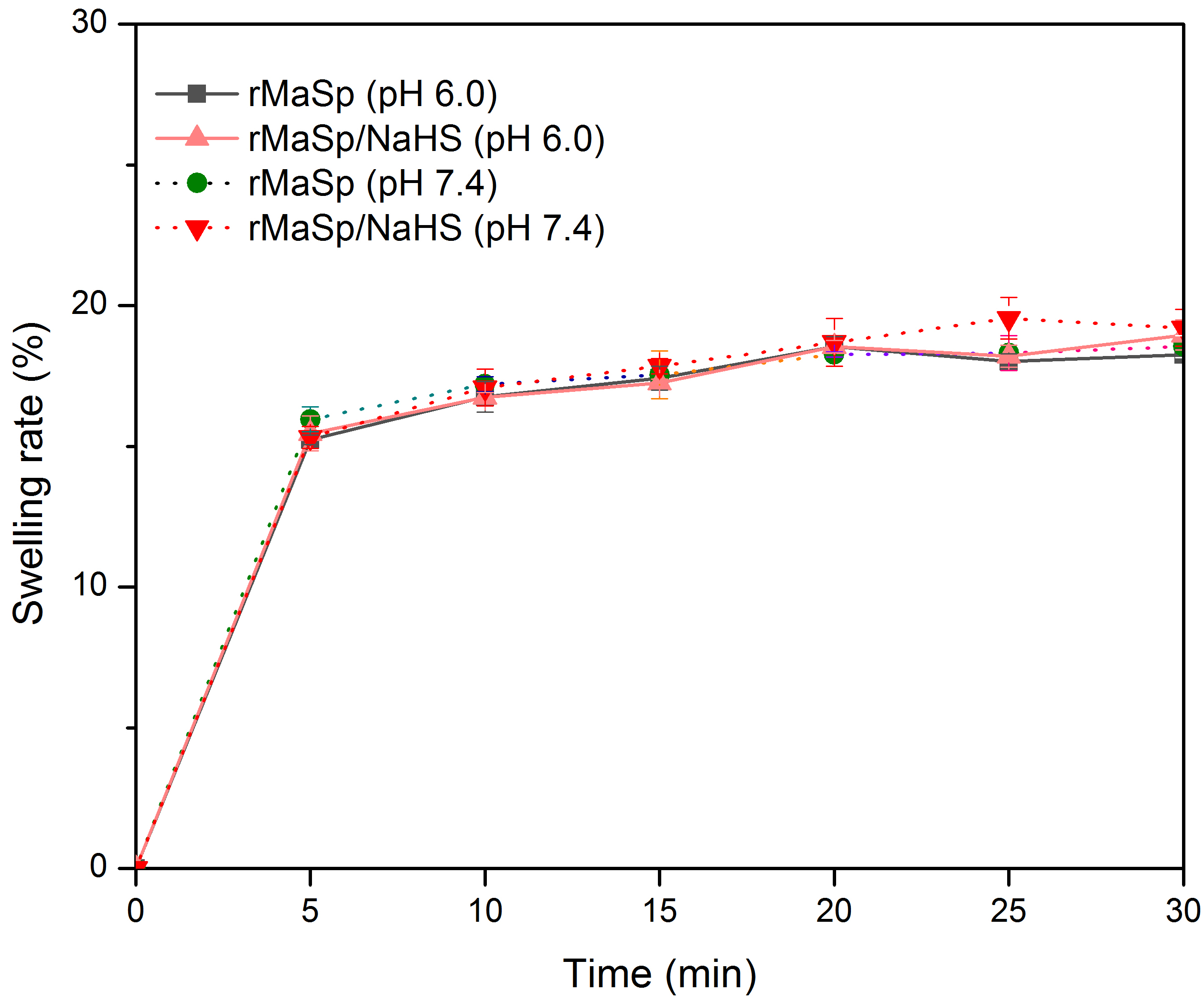
**

**Figure S4.** The water vapor transmission rate (WVTR) of the rMaSp/NaHS membrane with different concentration of NaHS.
